# Fit for purpose: German contributions to the new ICRP recommendations

**DOI:** 10.1007/s00411-025-01157-9

**Published:** 2025-12-04

**Authors:** Mandy Birschwilks, Omid Azimzadeh, Peter Scholz-Kreisel, Markus Eidemüller, Simone Moertl, Bastian Breustedt, Wei Bo Li, Maren Gruß, Christine Werner, Martin Steiner, Udo Gerstmann, Clemens Woda, Julius Vogt, Florian Gering, Katharina Stella Winter, Erik Björn Mille, Augusto Giussani, Goli-Schabnam Akbarian, Christiane Pölzl-Viol

**Affiliations:** 1 Federal Office for Radiation Protection, Ingolstaedter Landstrasse 1, 85764 Oberschleissheim, Germany; 2 Federal Ministry for the Environment, Climate Action, Nature Conservation and Nuclear Safety, Robert-Schuman-Platz 3, 53175 Bonn, Germany

**Keywords:** Detriment, Dose quantities, Optimisation, Societal aspects, Non-human biota, Impact assessment, Radiological emergencies, Medical application

## Abstract

The current international radiation protection system is based on the International Commission on Radiological Protection's (ICRP) policy proposal, Publication 103, issued in 2007. Recently, the ICRP has announced its goal to extend the discussion on radiation protection issues beyond the inner bodies, to engage the worldwide radiation protection community. A key step in this direction was the digital workshop "The Future of Radiological Protection" in October 2021 which initiated an in-depth international, scientific debate on the future design of the radiation protection systems. To contribute to this international debate, the German Federal Ministry for the Environment, Nature Conservation, Nuclear Safety and Consumer Protection (BMUV (now BMUKN)) and the Federal Office for Radiation Protection (BfS) in Germany hosted a workshop in Munich in November 2024, bringing together national and international experts to assess and prioritise key topics relevant to the future of radiological protection from a German perspective. The primary aim of the Munich workshop was to exchange scientific and regulatory perspectives in Germany, particularly with regard to the ICRP's “Fit for Purpose” process. Critical topics such as the revision of the justification principle, the refinement of the key criteria for radiation-related risk assessment and impact assessment were central themes of the discussions. This article presents the most important topics and recommendations discussed related to radiation detriment, dose coefficients, societal aspects, non-human biota, impact assessment, radiological emergencies and malicious events as well as the justification and optimisation of medical radiation applications.

## Introduction

In 2021, the International Commission on Radiological Protection (ICRP) began revising and reassessing the basic principles of radiation protection outlined in ICRP publication 103 (Recommendations of the International Commission on Radiological Protection – ICRP 2007), which represents the most important process for international radiation protection in the coming years. The ICRP assesses scientific findings for their relevance to radiation safety and develops fundamental radiation protection principles, with ICRP recommendations serving as the foundation for legislation, international standards and guidelines on radiation protection around the world.

In Germany, too, a large part of the legal regulations on occupational radiation protection and radiation protection of the population including medical applications are based on the recommendations of the ICRP, resp. their implementation in the European Union’s Basic Safety Standards (Directive 2013/59/Euratom). Significant changes to the existing international radiation protection system as potentially recommended by ICRP would have a fundamental impact on practical radiation protection worldwide. In the medium term, the national legislation on radiation protection in Germany would be affected by adjustments to the European directives. This shows how important it is to be part of the process from the outset and to help shape the international radiation protection regulations with a strong German voice. In order to effectively integrate the German position on radiation protection into this process, the German Federal Ministry for the Environment, Climate Action, Nature Conservation and Nuclear Safety (BMUKN^1^) and the Federal Office for Radiation Protection (BfS) jointly organised a national workshop with international participation in Munich in November 2024 titled ‘Fit for Purpose: The German contribution to the new ICRP recommendations’.

Radiation research and radiation protection is part of megatrends such as medical progress in diagnostics and therapy, digitalisation, climate adaption and the energy transition. German radiation protection has a high standard that must be maintained in the long term for the benefit of human health and the environment. At the same time, the radiation protection system is robust and it is necessary to assess where changes are really necessary to substantially improve the level of protection. The aim of the workshop was to highlight some of the areas in which improvements, optimisations or readjustments of radiation protection are required during the revision process from the German point of view or in which radiation protection already is successful and sufficient. Thus, the emphasis was on specific subjects rather than the radiation protection system as a whole.

The topics selected by the BMUKN and the BfS were radiation detriment, dosimetry, societal impacts, non-human biota, radiological emergencies and malicious events, as well as the principles of radiological protection: justification, optimisation, and dose limitation, alongside practical considerations, i.e. the necessity to balance continuity with the need to adapt to new requirements or scientific findings (impact assessment). The BfS has in-depth expertise on these topics and is also actively involved in the relevant work of the ICRP. This publication therefore provides recommendations on the topics which were examined during the workshop with both national and international experts.

The workshop brought together relevant experts from Germany and abroad, including scientists, policymakers, and radiation protection professionals, to discuss these issues and make recommendations. This paper presents the outcomes of these discussions at the November 2024 workshop in Munich and highlights the German contributions to the evolving international framework for radiation protection.

## Topics selected for in depth discussion at the workshop

### Future challenges for the radiation detriment—Cancer, heritable effects, cardiovascular diseases

Radiation detriment serves as a key measure of harm due to exposure with low dose of ionising radiation and is a central component of the system of radiation protection. The task of the detriment is, to calculate the potential harm of low dose ionising radiation to humans, regarding the incidence, the mortality and the loss of quality of life of a disease. With its focus on low dose ionising radiation only cancer and hereditary effects were included so far. Nonetheless, the last 20 years have seen significant advancements in science and medicine relevant to the detriment calculation. For example, better cancer survival rates and changes in baseline cancer risk emphasise the need for recalculation of the detriment. Recent scientific evidence underscores the potential for ionising radiation to induce a broader spectrum of health effects, including non-cancer diseases, and emphasises the necessity of integrating novel modelling approaches for the accurate assessment of cancer risk. To ensure that radiation detriment calculations are accurate and aligned with current knowledge, the following topics should be considered.

#### Methodological aspects of radiation risk models for ProZES and relation to the detriment

Within the development of the software ProZES^2^ for calculations of the assigned share of radiation for a given cancer (Ulanowski et al. 2020), several methods to estimate radiation-induced cancer risks were developed. Based on these results the following recommendations should be discussed for the recalculation of the detriment:

The current list of cancer sites for which the ICRP detriment is calculated contains several individual sites and a large group of remaining organs. The important remainder group is very heterogeneous and its risk estimates are not reliable. As an alternative, groups of cancer sites with functionally related cancers can be formed, leading to more biologically plausible risk estimates and a much smaller and less relevant remainder group.

The cancer risk models depend on attained age, age at exposure or time since exposure. In the last ICRP detriment calculation, an identical age dependence was applied for most solid cancer sites. All newer publications, e.g. from the Life Span Study (LSS) of the atomic bomb survivors from Hiroshima and Nagasaki, investigate the age dependence for each site separately. This is more plausible since the biological development of tumours differ largely between different cancer sites. There is no reason to assume that the age dependence of radiation risk should be the same or similar for different cancer sites.

With the method of Multi Model Inference (MMI) several risk models can be combined to a joint model, thus incorporating features from the individual models. While MMI has been used in radiation protection repeatedly, for the first time, ProZES made systematic use of MMI for all organs. In ProZES, MMI has proven to be an extremely helpful and effective tool. On the one hand MMI can be used to improve the estimation of uncertainties. On the other hand, it can also be used to create models that provide plausible risk values for many different exposure situations. Furthermore, information from different cohorts can be integrated.

#### A discussion on the inclusion of cardiovascular diseases (CVD) in low-dose radiation detriment calculations

While the term detriment as defined by the ICRP, specifically refers to the harmful stochastic effects of low-dose ionising radiation—such as cancer and hereditary damage—it did not account for circulatory diseases, including cardiovascular and cerebrovascular diseases.

Uncertainties in epidemiological data and limited understanding of biological mechanisms at doses below 1 Gy are cited as reasons for their exclusion. Moreover, CVD are considered as tissue reactions with a threshold dose of 500 mGy, which contrasts with the stochastic nature of detriment components (ICRP 2012, 2022b).

Recent epidemiological studies, however, suggest that even doses below 500 mGy may increase the risk of CVD. For instance, studies among atomic bomb survivors and occupationally exposed individuals indicate a heightened disease risk. In 2023, a comprehensive meta-analysis confirmed a dose-dependent increase in cardiovascular risks across a wide range of radiation doses (Little et al. 2023). Although the exact impact of doses under 100 mGy is not yet fully understood, these new data indicate a dose-dependent increase in disease probability, consistent with a stochastic radiation effect. Recent data associate CVD with clonal hematopoiesis of indeterminate potential (CHIP). In parallel, CHIP has been found to increase following exposures such as space radiation (Werneth et al. 2024), further suggesting a stochastic component in radiation-induced CVD. This emerging perspective could change our understanding of circulatory disease development and contribute to inclusion in radiation detriment calculations. Moreover, animal and human studies have provided valuable insights into the molecular mechanisms and pathological alterations of radiation-induced CVD at low doses (Tapio et al. 2021).

These recent scientific findings indicate growing evidence for an increased risk of CVD from low radiation doses that is not fully considered in current radiation protection guidelines. It may become necessary to include CVD in the detriment calculation and to consider consequences like recognition as occupational diseases and related compensation payments. This requires the development of risk models that account for individual differences such as gender and age, as well as the establishment of appropriate weighting factors for tissues. Identifying the most relevant target tissue representing the circulatory system is a critical yet unresolved question. Unlike cancer, where specific organs or tissues serve as targets for radiation risk assessment, circulatory diseases involve complex, systemic effects impacting the heart, brain, kidney and vascular system. Determining which tissue or combination of tissues best represents the circulatory system in radiation risk models is essential for improving dose estimates and understanding exposure–response relationships. Due to the importance of this topic, ICRP and UNSCEAR have established working groups to reassess the relevance of CVD following low-dose radiation exposure and examine possible inclusion in the detriment calculation.

To be prepared for future developments, strategies for integrating these risks into radiation protection should be developed promptly. However, it is important to note that including CVD with significant weight in radiation detriment calculations may lead to changes in dose limits. A change that consequently may require more cautious exposure guidelines to mitigate both cancer and non-cancer risks. The inclusion of CVD in detriment calculations is well motivated by recent findings; however, if full incorporation remains challenging, alternative approaches should be explored to address CVD risks more effectively in radiation protection strategies. One possibility, although far-reaching, would be the replacement of the current detriment model with another approach, such as the Disability-Adjusted Life Year (DALY), which has already been discussed within the scientific community.

#### The pros and cons of considering hereditary effects in detriment calculation

The hereditary effects of ionising radiation encompass all radiation effects that are transmitted to offspring through inheritance following parental exposure and may manifest in the next generation. The radiation exposure occurs pre-conceptionally; thus, irradiations of the fetus in utero are not considered hereditary effects. The inclusion of hereditary effects in the detriment was already considered with the introduction of the concept in radiation protection in 1977. This consideration was based on early biological observations of hereditary changes following radiation exposures in animal experiments. Unlike the radiation-related risks of cancer, there was no suitable data available on the risk of radiation-induced genetic diseases in humans (ICRP (2007), A11). Therefore, the calculation of detriment from hereditary effects was carried out indirectly using a "Baseline Frequency" and the "Doubling Dose", which is the radiation dose at which twice as many mutations occur in the genome as would be expected naturally from spontaneous mutations.

The data currently used in the calculation of "Baseline Frequency" primarily stem from studies conducted between 1970 and 1990, which are based on analyses of individual national registers (e.g. from Hungary) or individual national studies (e.g., from British Columbia, Canada). National differences in the definition or recording of hereditary diseases and ethnically based differences in the occurrence of hereditary diseases were not taken into account. Additionally, certain assumptions were made to estimate disease rates based on the scientific understanding at that time, which are now considered outdated. Data for the “Doubling dose” came from mouse experiments and were fitted to human observations using a correction factor.

But even new epidemiologic and biologic studies fail to prove the existence of radiation induced hereditary effects in human. There is still no data available to calculate valid risk estimates for hereditary effects after parental exposure to ionising radiation. Nonetheless, there is still a large social fear of possible hereditary effects, which manifests in phenomena like reduced birth rates after radiological accidents or psychological distress of expecting mothers due to exposure to ionising radiation. These anxieties have to be addressed in radiation protection.

The radiation detriment is based on risk estimates and because, even after intense research, there are no risk estimates showing a significantly increased risk for hereditary effects available, it is worth considering, whether these fears/concerns should be addressed in a more appropriate way than it is done by now.

#### Discussion results for detriment

Within the workshop discussions, there was broad consensus among the participants that there is a significant need for a considerable revision of the detriment. However, there is an intense discussion on the complexity of detriment calculation. While there is a scientific urge to implement new endpoints or methods into detriment, there is a loud call for a reduction of complexity to reach a better understanding and acceptance of the radiation detriment in the radiation protection community.

For acceptance of the detriment it is of paramount importance that its calculation is based on state-of-the-art scientific methods and models. The calculation methods should be transparent and the results robust against changes in underlying assumptions. Future re-evaluation of the detriment should include a critical revision of the cancer sites, or group of cancer sites, for which the detriment is calculated. The dependence of risk on age at exposure and attained age should be studied carefully for all groups. As worker cohorts become more and more informative, they should be integrated with risk assessment from the atomic bomb survivors. The method of multi-model inference can serve as important tool for this endeavour. The detriment calculation should include an evaluation of uncertainties. An open software program for the detriment calculation would be helpful for the broader radiation community and can improve transparency of the detriment calculation.

The current way of including hereditary effects into the detriment is inconsistent to the risk-based calculation of cancer detriment and leads to additional complexity and intransparency of the calculation of the detriment. Furthermore, hereditary effects add only minor values and would only have small impact on the detriment. Therefore, its exclusion would only have small impact on the weighting factors for the effective doses. Exclusion of the hereditary effects will reduce the complexity of calculation significantly and can largely increase the scientific rigorousity of the radiation detriment. Nonetheless, due to the societal anxiety of possible hereditary effects, we recommend to find a more suitable way to address those in radiation protection rather than in the detriment. A possible solution might be dose constraints in the procreative phase of life, comparable to dose restraints for minors or pregnant women.

At the same time, given the emerging evidence of circulatory diseases following low-dose radiation exposure, it seems necessary to develop methods to incorporate the risk of circulatory disorders in the assessment of detriment, with the same level of rigour as for cancer risks. Even if many questions remain unanswered, early engagement with these issues is highly recommended.

Future efforts should focus on updating baseline references for the incidence, mortality, and lethality of different subtypes of circulatory system diseases. Furthermore, the calculation of radiation detriment for CVD requires identifying the most relevant target tissue representing the circulatory system, a critical yet unresolved question. Determining which tissue or combination of tissues, best represents the circulatory system in radiation risk models is essential for improving dose estimates and understanding exposure–response relationships. Addressing these challenges is crucial for developing a more comprehensive and scientifically robust framework for incorporating circulatory diseases into radiation detriment calculations and assessing radiation-related health risks. This will require interdisciplinary research that combines epidemiology and radiobiological studies to gain a deeper understanding of the circulatory system's radiation response and accurately integrate it into detriment calculations.

#### Summary

Radiation detriment serves as the scientific basis for the radiological protection system; however, the system is complex and subject to ongoing critical discussions. The suggested updates and changes for calculating the radiation detriment can help to keep the detriment “Fit for Purpose” and address scientific and societal needs appropriately.

Emerging evidence challenges the current inclusion of hereditary effects and the exclusion of non-cancer health effects, such as CVD, in detriment calculations. Due to the deep impact of these changes on the bases of radiation protection an intense discussion of the issues and a broad acceptance within the scientific radiation protection community is necessary.

Efforts should be made to further develop the radiation detriment into a transparent tool that aids in estimating all radiation-related health effects. This requires a careful discussion of potential simplifications and a deep consideration between necessary improvements and additional, potentially less relevant complexities to ensure a balanced and evidence-based approach.

## Dose coefficients, quantities and monitoring.

### Introduction

Dosimetry is a key element in the system of radiation protection, because doses as a measure of the exposure to radiation form the basis of the assessment of risks. Without the proper measurement of doses, the quantification of exposures as the basis of the selection and implementation of radiation protection measures as well as the evaluation of their efficiency is not possible. Calculations and measurements of dose are daily business of the practitioners.

Based on the physical quantity absorbed dose, which gives the amount of energy (J) deposited per mass (kg) of material, ICRP derived dose quantities which consider radiobiology using weighting factors. The main quantity used in radiological protection is the effective dose, which is sex- and age-averaged and is used for the management of stochastic risks of radiation exposure. This use includes the setting of dose limits, optimisation of exposure by means of minimising resp. reducing effective dose and demonstration of compliance by monitoring of effective dose. The current general ICRP recommendations given in publication (ICRP 2007) updated the definition of dose quantities by providing a new set of weighting factors, while mostly maintaining the numerical values of dose limits. The new set of weighting factors required a recalculation of reference values of conversion factors (external exposure) and dose coefficients (internal exposure), which were updated in several publications following the 2007 recommendations (ICRP (2010),^2013^,2015b,2016b,2017b,2019a,2019b,2020b,2022a). This ongoing work included the application of updated reference computational phantoms (ICRP 2009a, 2016a, 2020a, 2020c, 2023).

### Implementation of operational quantities (ICRU 95)

By its definition effective dose as protection quantity cannot be measured. Thus, ICRU (International Commission on Radiation Units and Measurements) developed operational quantities (ICRU 1985, 1988, 1992, 1993), which are measurable point quantities and are conservative estimates of the protection quantities. The monitoring of radiation exposures by measurements is based on these quantities. Many devices (e.g., dosimeters), which give a reading in the operational dose quantities, have been developed and are in use. The devices and the algorithms used by the monitoring services to evaluate the measurements have been optimised over decades. A very high level of quality in the accuracy of the measurements and a good harmonisation of practices has been achieved in this time, which for example can be seen in the results of international intercomparison exercises (IAEA 1999, 2007a, EURADOS Reports 2021-05, 2021-06, 2022-01 ,2022-01, Böhm and Cruz Suarez (2001). These developments generate trust in the monitoring and the radiation protection practices.

**Fig. 1:**
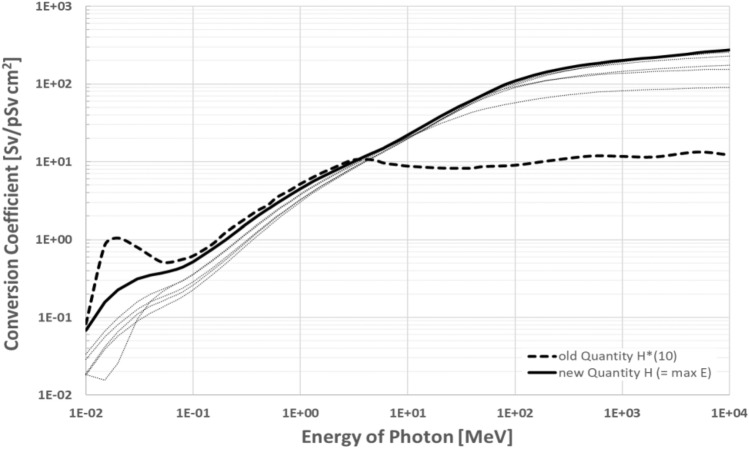
Fluence to effective Dose Conversion Coefficient for old and new quantity for area monitoring. Dashed lines give the coefficients E for different reference irradiation geometries. The figure was plotted using data from ICRP Publication 116 (ICRP 2010) and Pellicioni (2000)

One major change that needs to be considered carefully in the coming revision of the ICRP general recommendations is the updated definition of the operational quantities, which was published in the joint report of ICRU and ICRP (ICRU Report 95 (ICRU 2020)). In this report a major change in the concept of the definition of the operational quantity was introduced by directly using the effective doses as calculated in the ICRP reference phantom for the definition of the operational quantities. This resolves the problem of underestimation of the protection quantity for higher energies (i.e. photons > 10 MeV), when using the ICRU sphere in the definition of the quantity. The issue of overestimation for the very low energies (i.e. photons < 50 keV) is also resolved by this concept. Figure 1 illustrates the conversion coefficient for photons from flux to the operational quantities for area monitoring (old: H*(10), new: H = max(E_x_)) together with the conversion coefficients for the different irradiation geometries E_x_). Data for the new quantity and the irradiation geometries were taken from ICRP publication 116 (ICRP 2010). Data for the old quantities were taken from a publication of Pellicioni (2000), who calculated the conversion coefficients and extended their range. Note that in ICRP publication 74 (ICRP 1996) values for H*(10) are only given up to E = 10 MeV.

However, the currently well-established dosimeters, which are designed to meet the requirements (e.g. resemble the response and energy dependence) of the recent operational quantities, will require a re-design that is time consuming and costly.^3^ A lot of dosimeters, which are now in use, would need to be replaced. Some re-designed dosimeters might even require the use of additional detectors, which will increase their complexity and ultimately the costs. The requirements on accuracy of dosimeters might need to be relaxed as consequence of envisaged issues in the re-design of the dosimeters. It is questionable if all dosimetry services (esp. in the less developed countries) will be able to shoulder these big efforts and instead will adhere to their recent systems. It can be envisaged that, driven by these reasons, not all countries might adopt the new quantities in their regulations which ultimately will be a loss of harmonisation and introduces new challenges for the comparison of dose values and the exchange of workers across borders.

The direct coupling of the operational quantity to the protection quantity introduces a dependency on the weighting factors used in the effective dose. If in a revision of the general recommendations ICRP, based on discussion and re-evaluation of the concept of detriment, will change the values of tissue weighting factors w_T_ and ultimately the effective dose, the theoretical basis of the new definition of the operational quantities will then automatically change as well. The response of dosimeters, which are designed to meet the definition of ICRU Report 95 might in this case no longer be able to estimate effective dose properly. A concept to implement a stable and robust definition of the operational quantities needs to be included in the next general recommendations.

It needs to be thoroughly discussed whether the benefits of the new quantities outweigh the efforts required to implement them into practice. The gain in level of protection needs to be checked. Both are summarised in the following Table 1. If the new operational quantities as described in ICRU Report 95 are implemented the readings in measurements of low energy photons which are encountered in many medical applications, will decrease by half. It can be expected that the monitored staff might misinterpret these as real “lower doses” which could in turn lead to a reduced awareness and implementation of protection measure (e.g. a reduction of shielding). This could lead to an increase in real doses, which effectively reduces the level of protection and is against the ALARA culture. Additional education of staff will thus become more important in future.

**Table 1:** Benefits of and efforts required for implementing the new operational quantities.

Benefits	Efforts
Extension of radiation types and energy range in definition of operational quantities	Update of dosimeters required, leading to more complex dosimeters and/or the use of multiple dosimeters. High costs for updating the systems
Elimination of overestimation for lower (< 50 keV) energy photons and underestimation for higher energy (> 10 MeV) photons	Definition of quantities is indirectly dependent on detriment and is thus subject to change of basic parameters such as w_T_
Conceptual harmonisation of protection and operational quantities	Quality and harmonisation of individual monitoring practices achieved with many years of work might decrease
Use of absorbed doses for tissue reactions	Loss of awareness by lower readings of dosimeters

### Summary

In the process of updating the ICRP general recommendations, changes in the detriment will directly influence the definition of effective dose by changes in weighting factors and identifying new target tissues to be considered. ICRP models are ready to include these, however a close cooperation of the respective committees 1 and 2 of ICRP is required to update the dose coefficients and risk models.

ICRP should go into a dialogue with the stakeholders (here especially the individual monitoring services (IMS)) and keep the applicability of the recommendations (here the adoption of new operational quantities) in mind as the impact of these changes to the daily routine of radiation protection practitioners worldwide is expected to be large. It should be discussed whether the huge effort required to implement new dosimetric systems is justified in terms of an added value for reaching the goals of dosimetry, esp. when the large uncertainties in personal dosimetry are considered.

## Societal aspects in radiation protection.

Although the human factor and agency are mentioned in the objectives of the recommendations, and the relevance of considering social impacts is acknowledged (ICRP 2007, p. 41–46), these aspects are not implemented in the actual recommendations. This is due to the ICRP’s exclusive focus on natural sciences. An evidence-based understanding of human behaviour and perception in the context of radiological protection also requires expertise from social science and humanities (SSH) research. As SHARE (European Platform for Social Science and Humanities in ionising radiation REsearch) has already noted, an insufficient integration of these disciplines into radiation protection projects can lead to a range of issues, like “*unresolved risk management decisions or confusion in public opinion regarding radiation protection issues, e.g. low public acceptance of decisions related to radioactive waste management, high risk perception of certain uses of ionising radiation (e.g. food sterilisation), lack of radon awareness, weak connection between emergency management plans and practice, security challenges, and deficiency of informed consent in the medical field*” (SHARE, n.d.; retrieved August 08, 2025, from https://www.ssh-share.eu/background/).

To refine the radiation protection framework, it is important to engage more effectively with societal trends, individual behaviour, and stakeholder dynamics. Despite the sound scientific basis of the ICRP framework in scientific disciplines such as physics, biology, dosimetry, epidemiology, practical challenges often arise when integrating the system into various societal systems. However, the Fit for Purpose process offers the opportunity to address societal issues of radiation protection in a systematic way. The recommendations could significantly benefit from placing a stronger emphasis on the societal perspective and systematically integrating it into the design and implementation of protection measures. This would not only improve the effectiveness of measures but also enhance public acceptance and trust in the institutions making decisions on protection measures (Perko et al. 2019).

### Interdisciplinarity is key

When examining the human element in radiation protection more closely, several challenges unveil: the challenge to create awareness and an understanding of radiation risks within the public, followed by understanding the publics’ perception of risks as well as their concerns, and engaging different stakeholders in complex decision-making processes (Cho et al. 2014). These are just a few areas in which a deeper understanding of human perception and behaviour and their integration into a holistic radiation protection system can significantly advance radiation protection. Moreover, being aware of the social factor in radiation protection, communication and dialogue within and about the radiation protection system can be enhanced.

The same applies to ethical aspects, which up to now have been addressed in different ICRP task groups and publications. The foundation for considering ethical aspects in radiological protection was laid in ICRP publication 138 “Ethical foundations of the system of radiological protection” (ICRP 2018). Since then, task group 109 “Ethics in Radiological Protection for Medical Diagnosis and Treatment” (ICRP Publication 157 (2024) “Ethics in Radiological Protection for Patients in Diagnosis and Treatment”), Task Group 114 “Reasonableness and Tolerability in the System of Radiological Protection”, and Task Group 129 “Ethics in the Practice of Radiological Protection” have been dealing with ethical aspects in different radiological protection fields. The fundamental and universally valid ethical aspects must also be an integral part of the basic radiological protection concept.

As highlighted by the aforementioned examples, the aspects associated with considering the human element in radiation protection are important and diverse at the same time. Accordingly, the ICRP should enhance comprehensive interdisciplinarity in its task groups and committees by integrating social sciences (at the very best even humanities) alongside natural sciences when societal aspects are relevant. Each ICRP committee should assess the societal implications of its work, which could include topics such as communication, participation, mental health or ethical aspects. Particularly, Committee 4 (Application) should consider including social science experts to put the ICRP recommendations into societal practice. The ICRP should incorporate the societal handling of radiation and radiological protection, the importance of risk perception, and communication into its recommendations. Given cultural differences across nations, mostly general recommendations and guidelines should be given to ensure universal applicability.

To expand the ICRP's network with experts from other disciplines, programs like the ICRP's mentee program are an effective tool. By incorporating mentee positions, the ICRP can address specific societal issues and engage young scientists from fields like social sciences early in their careers. Closer collaboration with social science consortia, such as SHARE, is also beneficial, as these consortia can provide advisory support on specific societal issues (SHARE 2024).

Two ICRP examples support this approach: 1) The mentee program was used by TG120 (Radiological Protection for Radiation Emergencies and Malicious Events) under committee 4 to recruit communication experts who integrate communication recommendations in radiological emergencies and current societal developments into the recommendations for radiological emergencies in an interdisciplinary way. 2) The ICRP Main Commission has recognised the special role of effective communication in the strategic direction and public perception of the organisation. To address this, they have established a working group of practitioners with experience in communication and interdisciplinary backgrounds.

By embracing a holistic, interdisciplinary approach that incorporates social sciences and humanities, the ICRP can address two key opportunities to enhance the effectiveness of its recommendations. Firstly, its communication guidelines can become more evidence-based, aligning with the principles of communication science to ensure appropriate communication. Secondly, by integrating social perspectives, the ICRP can expand its health concept to include mental health considerations, benefitting the general population, emergency responders, and personnel.

### Evidence-based communication design

The Vancouver Call for Action has already emphasised the necessity of using plain language in communication with the public and decision makers to foster risk understanding, informed choices and an objective view on risks and benefits (Rühm et al. 2023). The ICRP has addressed this partly through ICRPaedia, an accessible reference in plain language. In Publication 103 the ICRP (2007) states that “advice of the Commission is aimed principally at regulatory authorities, organisations, and individuals that have responsibility for radiological protection” (p. 38). Even though the general public is not a specific target audience of the ICRP, the technical language style chosen for the publications is difficult to understand for the mentioned target groups as well.

Even though the use of plain language is an important prerequisite for effective communication, especially risk communication, it is by no means the only one. Effective risk communication is based on several key principles that ensure important information is conveyed effectively and with trust. This includes *comprehensibility*, ensuring that messages are clear and understandable for the target audience. *Communication at eye level* fosters trust and respect between the sender and receiver. *Competence, fairness, and empathy* are crucial for responding credibly and sensitively to stakeholders' concerns. *Honesty and openness* create transparency and allow for open dialogue. *Collaboration* with other credible entities enhances the message and increases credibility. Additionally, *attention to the needs of the media* is important for an impactful dissemination of information. Finally, *careful preparation and continuous evaluation* are essential to ensure the effectiveness of communication and to continuously improve it (Leidecker-Sandmann et al. 2025; IRPA 2020; Perko et. al. 2020; Pölzl-Viol 2022; WHO 2017). The accomplishment of these principles depends on the individual, situational and cultural context in which the communication takes place. Depending on the aim of communication some of the previous mentioned principles of effective risk communication are more important for the ICRPs purposes than others. Regardless the context, risk communication is generally understood to mean an “exchange of information and opinions about risks, risk avoidance, minimisation, and acceptance of risks” (BBK 2019, S. 46). This includes a process of mutual exchange of information and opinions about real, potential, or perceived risks, risk-relevant factors, and risk perception between (scientific) experts, risk managers (authorities), and the "public" (affected parties, interest groups, etc.). This definition already suggests that experts' risk assessments do not necessarily align with the perceptions of laypeople. Risk perception refers to the process of subjectively assessing a risk situation based on intuitive judgment, personal experience, and received information (e.g., through media), and can be influenced by characteristics of the situation, the damage, and the individual (Renn 2014). If risk perception is not taken into account when communicating risks, the communication efforts are likely to fall short and the intended objectives may not be achieved.

Even though the ICRP aims to foster risk understanding, informed choices and an objective view on risks and benefits (Rühm et al. 2023), its recommendations often overlook effective risk communication principles and the perception and actions of affected groups. The recommendations should include communication strategies for the targeted audiences, who in turn need to engage with affected groups to effectively implement the ICRP recommendations. Not only does the ICRP ensure that the content of its communication is based on evidence, which is a core principle supported by the thorough scientific efforts of its Task Groups, but the way communication strategies are developed and recommended should also rely on scientific findings. Otherwise, an effective implementation of the ICRP recommendations into societal practice is challenging. Concrete recommendations for using plain language should be integrated. Additionally, the ICRP's commitment to formulating the protection concept in simple language should be strengthened. This approach will enable the targeted audiences, who may not operate at the same scientific level, to be more effectively reached.

By systematically examining the socio-relevant context beforehand, communication recommendations can be tailored to the specific topics of a Task Group, while considering the interests and perceptions of relevant target audiences.

The advantages of incorporating evidence-based communication recommendations into the work of the ICRP should not be underestimated. When risk communication is effectively designed, it can increase attention and awareness, helping to close gaps in knowledge and fostering a more realistic assessment of risks. It strengthens motivation for protection by clearly communicating protective measures and highlighting the personal impact of risks.

Thus, the ICRP can ensure that its recommendations are not only theoretically sound but also practically viable and socially relevant.

### Mental health in radiation protection

ICRP Publication 103 outlines basic principles for appropriate radiological protection: “…the Commission's system of radiological protection aims primarily to protect human health. Its health objectives are relatively straightforward: to manage and control exposures to ionising radiation so that deterministic effects are prevented, and the risks of stochastic effects are reduced to the extent reasonably achievable” (ICRP 2007, p 41). This health concept focuses narrowly on physical health effects. According to the WHO, health is a state of complete physical, mental (psychological) and social well-being (WHO 1948).

Radiological emergencies like Chornobyl and Fukushima highlight that these catastrophes impose mental stress, affecting mental health, social well-being, and physical health (Bromet et al. 2011; Shigemura et al. 2020). Psychological stress symptoms are caused particularly by fear and uncertainty in relation to the effects of radiation. This emphasises the great importance of good and evidence-based communication, as outlined above. In addition to scientific findings on radiation effects, exposure and risk, good radiation protection depends on considering the public's awareness and protective behaviour, enabling informed decision-making in any exposure situation. Furthermore, protective measures as part of radiological emergency management, such as staying indoors, decontamination or evacuation, are stressful situations and can lead to psychological stress symptoms. Also, stigmatisation due to the fear of radiation and loss of trust in emergency management bodies can pose mental stress (WHO 2020).

The range of consequences of mental stress is broad, such as depression, anxiety and panic disorders, grief, sleep disorders, psychosomatic complaints, lower assessment of personal health, increased cigarette and alcohol consumption, up to suicide. A review from the year 2019 on mental health consequences of the Fukushima nuclear disaster affirmed the consequences for mental health investigated in various studies (Shigemura 2020). Depending on the research method used, increased rates of non-specific psychological distress (8.3%-65.1%), depressive symptoms (12%-52.0%) and post-traumatic stress symptoms (10.5%-62.6%) were found. While not all effects are directly linked to radiation, the perception of risk, the social network, familiar surroundings and hostility from others also play an important role for mental wellbeing, the fear of radiation, stigmatisation due to radiation and insecurity are dominant factors. Important groups at risk for mental health impacts are for example first responders, mothers with children and clean-up workers. Further risk groups suffering from mental health burden in radiological emergency situations are described in WHO 2020. The OECD NEA’s guidance (2024) provides specific recommendations for reducing the psychosocial impact of radiological disasters and to consider specific needs of vulnerable groups, mainly grouped within the “5C” Coordination, Communication, Capacity Building, Community Engagement and Core Ethics. With respect to the ICRP’s Fit for Purpose process, four main aspects are considered as important: Mental health und psychosocial wellbeing have to be considered as part of the concept of health in radiological protection (1); informing about mental health consequences should be part of protection system (2); improvement of risk and crisis communication to improve the sense of the public of safety and trust (3); strengthening the public’s self-help-ability can contribute to reducing mental health burden (4).

The ICRP has various options to more strongly take a holistic health concept into account:

1. A commitment to the holistic concept of health, which is also respected by the ICRP, with reference to the fact that the competence of the ICRP lies in consideration of the deterministic and stochastic effects of ionising radiation and that aspects of the impairment of mental health cannot be addressed. Reference can be made to relevant documents.
2. Specific findings on the psychosocial effects of radiological emergency situations are included in the ICRP recommendations and supplemented by specific instructions for action. As this is the basic radiation protection system of the ICRP, comprehensive explanations are out of place. However, specific publications on radiological emergency protection should consistently consider mental health and psychosocial needs.

### Summary

Health is more than physical integrity. As the ICRP recommendations are the basis for standards and legislation worldwide, it is important that this important document i) expands the concept of health and describes psychosocial effects; ii) provides concrete recommendations for action to take social aspects into account and iii) includes evidence based recommendations for appropriate communication of radiation risks.

The ICRP should strive for holistic interdisciplinarity by incorporating social sciences and humanities into its scientific expertise. However, the active recruitment of experts from other disciplines requires personnel resources from the ICRP. This is particularly true since radiation protection can only be considered a niche topic in the context of social sciences and humanities. While networks of experts from these disciplines, such as SHARE, already exist, the number of experts is limited. Active networking with individuals and institutions engaged in other areas is necessary to expand the interdisciplinary network. In this regard, the work of the ICRP’s Communications Working Group can be particularly beneficial.

If the ICRP decides against the proposed solutions, it is necessary to reflect on the limitations of its own expertise. Nevertheless, it remains obligated to acknowledge these aspects as relevant and to direct attention to appropriate institutions and sources that provide recommendations. However, it should be noted that such an approach is no longer considered contemporary as there is a general consensus that a holistic approach to problem-solving is preferable.

Incorporating the ICRP’s recommendations on interdisciplinarity, evidence-based communication, and Mental Health and Psychosocial Support (MHPSS) into national policies requires national institutions to have expertise in these areas, either by developing their own experts or by acquiring external expertise. While this implementation entails effort, it is feasible, particularly at higher organisational levels where institutions can collaborate to pool resources and develop joint solutions. Strengthening the Social Science and Humanities (SSH) domain across all scientific and policy levels would result in a consistently improved radiological protection framework, leading to more comprehensive and effective strategies.

## Non-human biota

### Introduction

The explicit protection of non-human biota against harmful effects of ionising radiation has emerged as a complementary concept in the radiation protection system during the past decades, addressing a previously existing conceptual gap in the radiation protection system and reflecting an increased awareness of environmental stewardship. Radiation protection of the living environment aims at preserving the health and functionality of ecosystems, i.e. stable populations of animals and plants, and maintaining biological diversity.

Despite international efforts to establish a dedicated approach to protect non-human biota, the importance of ionising radiation as an environmental stressor to wildlife has not yet been quantified in a systematic way. Non-human species are subject to various stressors, including food scarcity, diseases, pollutants (e.g., NO_x_, microplastics), climate change and direct human interventions in ecosystems.

Ionising radiation is an additional stressor but, to our knowledge, there is no evidence that ionising radiation has a significant impact on stable populations and biodiversity, apart from severe emergency exposure situations. Radiation protection of non-human biota is built upon the assumption that ionising radiation is a relevant stressor for the living environment.

The Treaty on the European Atomic Energy Community (Euratom) refers only to radiation protection of humans, whereas Directive 2013/59/Euratom (EC 2014) implicitly includes environmental radiation protection, since it may impact human health in the long term (Article 2). (Non-binding) recital 27 therefore calls for a policy to protect the environment against the harmful effects of ionising radiation. This recital also states that environmental criteria based on internationally recognised scientific data, such as published by the ICRP among others, should be taken into account.

In 2016 the German Commission on Radiological Protection published the recommendation “Protecting the environment in the context of radiation protection”, taking into account the concepts of conventional environmental protection, the results of various EU projects, the at the time state of discussion of ICRP and a concept developed by BfS (SSK 2016).

Since the ICRP recommendations consider explicit protection of non-human biota an essential element of the radiation protection system and current ICRP publications provide a complete toolbox for calculating and evaluating the exposure of the living environment, the BfS used the pertinent ICRP publications to develop a generic approach to environmental radiation protection.

### The ICRP toolbox

The ICRP took its first step towards a systematic approach to the protection of non-human biota in the 2000s, with its recommendations evolving over time. In 2007, the ICRP recommended for the first time the explicit demonstration of environmental protection against ionising radiation in ICRP Publication 103 (ICRP 2007) and thus expanded the prior thesis that the protection of humans ensures adequate protection of non-human biota. To operationalise the approach to environmental radiation protection, ICRP Publication 108 (ICRP 2008a, b) introduced the concept of Reference Animals and Plants (RAP), providing a structured methodology aligned with existing environmental impact and risk assessment frameworks. The 12 RAPs are assumed to sufficiently cover the various groups of organisms (mammal, bird, amphibian, fish, insect, plant, etc.) and their habitats (marine, limnic, and terrestrial) with regard to radiation protection of non-human biota. For dosimetric purposes the RAPs are represented by simplified geometrical bodies (like spheres and ellipsoids). With ICRP Publications 114 (ICRP 2009a, b), 124 (ICRP 2014), 136 (ICRP 2017a) and (ICRP 2021), the ICRP provides a comprehensive toolbox for calculating the weighted absorbed dose rates for 12 RAPs and 75 radionuclides, including radiation weighting factors, concentration ratios for the radionuclide transfer from environmental media to reference organisms, and dose conversion factors.

The benchmarks for assessing the weighted absorbed dose rates of the reference organisms are the Derived Consideration Reference Levels (DCRL) as introduced in ICRP Publication (ICRP 2008a, b). The DCRL values span an order of magnitude each, reflecting the different radiation sensitivity of the organism groups and the existing uncertainties at low dose rates based on the current available data. Since the total dose rate is decisive for adverse effects of ionising radiation on the living environment, the DCRL ranges were introduced by the ICRP as total exposure, i.e. as the sum of natural and civilisational dose rate contributions. Nevertheless, the ICRP recommends in ICRP Publication 124 applying the DCRL values only if there is an “incremental environmental exposure of significance above the natural background” (ICRP 2014). The conceptual inconsistency that arises from neglecting the natural background is considered a major shortcoming.

It should be noted that other methods for determining the exposure of the living environment are also available. In Europe, the ERICA Assessment Tool (ERICA Consortium 2023) is widely used; in the USA, RESRAD-BIOTA (ANL 2016) is the standard tool specified by the US Environmental Protection Agency (EPA).

### Explicit consideration of the natural background

The systematic and consistent treatment of natural background is a challenging task. On the one hand, adverse effects of ionising radiation on non-human biota arise from the total exposure, which includes both natural and civilisational contributions. Natural background radiation, however, varies widely across regions and ecosystems, which is why an explicit, location-specific calculation of the natural contribution to exposure is inappropriate for regulatory purposes and expensive. On the other hand, regulatory frameworks usually focus solely on the civilisational increment. Hence, it is necessary to bridge between the scientific basis and the regulatory needs.

Although the natural contribution might dominate the exposure of reference organisms, the ICRP recommends comparing only the civilisational exposure increment with the DCRL values without further scientific explanation (see, for example, ICRP Publication (ICRP 2014)). The exposure of the living environment is thus an example of how the a priori exclusion of the natural background makes the scientific rationale difficult or even impossible. The BfS suggests initially explicitly including natural exposure contributions and then deriving regulatory implications, notably an acceptable civilisational exposure increment, through robust scientific analysis. A structured conceptual approach to consider natural background is presented in Sect. "Summary".

### The BfS concept and its application

As has already been discussed, the systematic treatment of the natural background is a challenging task. There is a risk of trying to protect the environment from itself if the background exposure level is found to be above the protection threshold (Brownless 2007). However, there is no need for action if an ecosystem is (largely) unaffected by man, irrespective of the type and impact of any potentially harmful substance. Applying this basic principle to radionuclides and ionising radiation in general, any human action to reduce the exposure of living organisms is not justified in an ecosystem with negligible human impact.

In order to conceptually include natural exposure of biota in a coherent and transparent way, the BfS developed a two-step approach for chronic exposure situations, exclusively considering the 12 reference organisms and the 75 radionuclides for which the ICRP provides all necessary data. In the first step, the total weighted dose rate to reference organisms, i.e. the sum of natural and civilisational contributions, is considered. If it is below the upper values of the DCRL bands for each reference organism, non-human species are regarded adequately protected according to the current knowledge of the dose–effect relationship for reference organisms. If the total weighted dose rate exceeds this threshold for at least one reference organism, the exposure increment arising from human activity should be considered in a second step. It may a priori be zero in case of negligible human impact or should not exceed an acceptable level.

The choice of an acceptable civilisational increment is a delicate task, especially in situations with a high natural exposure level. For planned exposure situations or future practices that may enhance the contamination of environmental media, the civilisational exposure increment should be limited in consideration of the ALARA principle. The BfS proposes striving not to exceed an incremental dose rate of 10% of the upper values of the DCRL bands. For existing exposure situations, such as former mining or industrial processing of radioactive material, the civilisational exposure increment should be the outcome of a mandatory optimisation process with priority of human radiation protection. In emergency exposure situations, all resources are expected to be used for human radiation protection, with environmental radiation protection being of subordinate importance. The two-step approach suggested by the BfS demonstrates that it is sufficient to regulate the civilisational exposure increment to reference organisms even if the natural background is explicitly taken into account.

As a pragmatic and fit-for-purpose approach, the BfS suggests demonstrating adequate protection of the living environment in a generic way instead of case-specific evaluations. It is expected to work in densely populated areas with a sophisticated radiation protection system for members of the public such as Germany. To protect members of the public against ionising radiation, dose limits apply and compliance with these limits has to be demonstrated in authorisation and notification procedures. The dose limits for human radiation protection implicitly set maximum contamination levels of the environmental media soil, (surface) water and air. From the maximum contamination levels of the environmental media that are compatible with the dose limits for members of the public, the civilisational contribution to the weighted absorbed dose rate of the reference organisms is then calculated using the ICRP toolbox described above. Finally, the calculated exposure is compared with the dose criteria proposed by the BfS for radiation protection of the living environment (10% of the upper values of the DCRL ranges). In this way, it can be generically checked whether compliance with the dose limits for members of the public also ensures radiation protection of the living environment by limiting the contamination levels of environmental media. These generic model calculations ensure radiation protection of the living environment in accordance with the state of the art in science, while minimising regulatory efforts.

### Fit for Purpose process of the ICRP and future activities

The ICRP’s Fit for Purpose process for the radiation protection of non-human biota is an ongoing effort which focuses on refining and adapting its framework in response to scientific advancements, regulatory needs, and practical considerations. From the perspective of a regulator, the most pressing issue is providing evidence that ionising radiation is a relevant stressor for the living environment compared to other natural and anthropogenic stressors. At present, the ICRP framework for environmental radiation protection is built upon the assumption that this is the case.

As part of the process, ICRP Task Group 99 intends to broaden the RAP concept to taxonomic groups and develop a methodology for deriving revised DCRL values via statistical models (ICRP 2017a, b, c). A further goal is to integrate radiation protection of the living environment more closely into interdisciplinary environmental and ecosystem studies in order to be able to assess the influence of ionising radiation in interaction with other environmental stressors. According to Clement et al. (2021), future recommendations may also go beyond the initial focus on biodiversity and the functioning of ecosystems, considering managed ecosystems, domesticated species and ecosystem services. Radiation protection of pets and livestock, however, is irrelevant for preserving stable populations, biodiversity, and ecosystem functionality und should therefore conceptually be distinguished from environmental radiation protection.

The Fit for Purpose process of the ICRP would greatly benefit from providing guidance on how to apply ICRP recommendations and paying attention to practical aspects. The challenge will be to translate complex scientific findings into recommendations that are easy to apply in regulatory and operational terms.

### Summary

The current ICRP recommendations provide a comprehensive toolbox for calculating and evaluating the radiation exposure of the living environment. Based on these recommendations the BfS developed a fit-for-purpose approach to environmental radiation protection, explicitly including the natural background and demonstrating that it is sufficient to regulate the civilisational exposure increment of reference organisms. Furthermore, the BfS proposes generic calculations to demonstrate that the living environment is adequately protected in densely populated areas with a sophisticated radiation protection system for members of the public. It is based on the idea that the dose limits for members of the public implicitly set maximum contamination levels of the environmental media, which in turn limit the civilisational contribution to the exposure of reference organisms. The results of these generic calculations are expected to show that further legal regulations to protect non-human biota against ionising radiation are not necessary in Germany.

The BfS supports the further development and refinement of the radiation protection system of the living environment with regard to the scientific knowledge, for example by taking into account sensitivity distributions for deriving DCRL values or the latest advancements in wildlife dosimetry as done by the IAEA coordinated research project K41023 (IAEA 2022). However, one of the ICRP's main tasks from a regulator's perspective will be to translate these complex scientific relationships into easily implementable recommendations that require as little effort as possible. Moreover, the BfS recommends quantifying the relevance of ionising radiation compared to other environmental stressors in chronic low-dose exposure situations.

## Radiological emergencies and malicious events

### Introduction

Radiological emergencies and malicious events do not only comprise large-scale emergencies, such as severe accidents in nuclear power plants, but also malicious events induced by perpetrators purposely to harm personnel and the environment. The number of exposed individuals (workers, members of public, first responders) in the various scenarios can vary from only a few to tens of thousands. Tools for rapid assessment of the radiological situation and planned-in-advance protective actions are needed to mitigate the impact on human health. Such kind of radiological and nuclear incidents pose specific challenges to emergency preparedness and response but also to the system of radiation protection as a whole, which must be addressed.

#### The need for dosimetric tools, appropriate quantities, recommendations and advice

ICRP has already taken action to close certain gaps in nuclear and radiological emergency preparedness and response by initiating two task groups on the subject: ICRP Task Group 120 (radiation protection for emergencies and malicious events (including nuclear detonations)) and Task Group 112 (Emergency dosimetry).

Task Group 120 was established to update and expand the scope of previous publications on the subject, based on the analysis of past emergencies, and will give recommendations for a wide range of nuclear and radiological emergencies that are not large-scale nuclear accidents. The advice will be guided by the four goals of radiation protection: to avoid deterministic effects, to reduce stochastic effects as much as possible, to protect people and the environment and to cope with non-radiation effects (psychological, social & economic). Concerning the scenario of a nuclear detonation, a multilingual advice for the public was prepared (ICRP: https://www.icrp.org/page.asp?id=611#10MIN), contributions from individuals and organisation outside the task group were possible. This multilingual approach highlights the importance of transitioning scientific knowledge into practical guidance. A similar publication is available from the German Commission on Radiological Protection (SSK), with additional detailed information about the scientific background of the recommendations (SSK 2024). Further information can be found on the website of BfS (https://www.bfs.de/DE/themen/ion/notfallschutz/folgen/mensch/mensch_node.html).

Task group 112 was initiated because the current ICRP dosimetry system optimises protection against stochastic health effects for low dose occupational exposure, but is not optimal for protection against tissue reactions. In the case of localised external exposure or localised concentration of radionuclides in the body, high local doses, exceeding the threshold for deterministic effects leading to tissue reactions could be associated with low effective doses. But even as measure of radiation risk following emergency exposures, effective dose is of limited value. First it is a sex and age-at-exposure averaged quantity (ICRP Publication (2007)) and therefore does not reflect the significant variation in radiation sensitivity in the population. Second, a dose and dose-rate effectiveness factor (DDREF) is applied (ICRP Publication (2007)) to account for potentially lower risks after low dose/low dose rate exposures, a circumstance that is not necessarily given in emergency scenarios. Corresponding arguments can be found for the limitation of the use of the committed effective dose in the case of emergencies. The ICRP reference dataset will therefore be expanded to consider harmful tissue reactions (based on absorbed doses), stochastic effects and situation specific conditions. The datasets and tools will be specific for different emergency phases (preparedness, response and transition phase) and involved stakeholders. A potential issue of the planned task group output is that tissue absorbed dose could be recommended to be used both in the response phase—where especially for acute exposures effective dose indeed seems to be the inappropriate quantity – and (potentially) also in the preparedness phase, when planning for protective actions during an emergency. At present it is unclear how a single effective dose threshold value for e.g. evacuation or sheltering could be meaningfully replaced by a combination of organ absorbed doses. For thyroid blockage the dose criterium will have the same numerical value, whether it is reported in mGy or mSv. While it can be expected that the “protective unit” mSv is at least basically known to decision makers and the public, the physical measurand “mGy” is largely unknown. This requires additional explanations, which can lead to misunderstanding and confusion. An advantage of using absorbed doses is the possibility to estimate radiation risks more accurately, either for individuals after exposure for medical follow-up or for population groups based on dose prognosis in the pre-release phase (Walsh et al. 2019). In general, methods and approaches in emergency preparedness and response need to be balanced between scientific accuracy and applicability in operational practice (especially under the circumstances of an emergency situation).

#### The potential of biodosimetry and clinical outcome prediction tools

For the management of accidental and mass casualty radiation exposure scenarios at the clinical level certain requirements have to be met. A holistic diagnostic approach, consisting of the interplay of biodosimetry, physical dosimetry and clinical outcome prediction might be suitable for the medical management of radiological mass-casualty events. The approach is scalable to deal with patients ranging from a single one to a million. For clinical dosimetry/outcome prediction, the H-Module was developed to triage according to acute radiation syndrome (ARS) (Majewski et al. 2020; Port & Abend 2018). The module is based on blood count parameters and on a database with 1,000 patient cases. The module is fast (1–3 days), easy to use and to train. In this context it was ascertained that hospitals can provide 10,000 measurements per day. Biodosimetry can give guidance for individual medical treatment and offers the opportunity not only to deliver dose but possible clinical endpoints. The major European biodosimetry laboratories are organised in the RENEB^4^ network, with an estimated capacity of around 5,000 samples per week.

#### How to integrate the response to nuclear security events into a holistic CBRN approach

In the case of a malicious event to harm the public, it cannot be excluded that perpetrators would rely on using different agents (chemical, biological, radiological and nuclear—CBRN) simultaneously to maximise the harmful impact. For such a combined CBRN event, the challenge thus lies in the simultaneous occurrence of a nuclear security event and other security events. In this context the UvB-CBRN (UnterstützungsverBund CBRN) in Germany, an umbrella structure on the federal level to deal with CBRN events, can be seen as a good practice example to deal with an all hazard approach. The lead in this umbrella structure lies with the German Federal Police. Partner authorities are the German Federal Criminal Police Office, specific units of the German armed forces, Robert-Koch-Institute and the BfS, the latter as experts for radiological and nuclear events. The UvB-CBRN follows the all-hazard approach according to (IAEA 2007, 2024). For this purpose, e.g., Standard Operating Procedure (SOP) of common processes of the involved authorities are developed together, arrangements are made to meet the timescale for a response and interoperability of authorities is key. The readiness is improved and strengthened by technical improvements, collaborations in working groups, further training of all partners and lessons learned from exercises and deployments. The all-hazard approach means acting, exercising and deploying together before and after an incident.

#### Radiation protection in case of a nuclear detonation scenario

Both, the US and Russia possess large arsenals of strategic and non-strategic nuclear weapons. Additional nuclear weapon states exist (e.g. People's Republic of China, United Kingdom, France), with however much lower amounts of nuclear weapons. In the case of deployment of a non-strategic nuclear weapon, the extent of the direct (lethal) weapon effects depend on the explosive yield (in equivalent of kilotons of TNT). For smaller yields, radiation effects (e.g. 5 Gy isoline) reach further in distance than thermal or overpressure effects, for large yields the order is reversed. Although direct effects are severe, the induced consequences are still localised. Effects due to fallout have a much larger range and are thus significantly important for radiation protection measures. Efforts are therefore being undertaken e.g. at the national level and in an European project to improve the atmospheric dispersion modelling capabilities for nuclear detonations for a more accurate prognosis of the radiological consequences due to fallout (https://pianoforte-partnership.eu/predict/). This will also include advice and communication material for the public, which should be coordinated with the current ICRP efforts mentioned before.

#### Summary

A number of initiatives and approaches within and outside the ICRP community have been described that, as diverse as they may appear, have the common goal of improving the current radiological and nuclear emergency preparedness and response capabilities. The use of tissue absorbed dose for all dose criteria in the different phases of a radiological or nuclear emergency seems to be more appropriate from a scientific point of view but needs to be also critically assessed from the operational point of view. Future ICRP publications and recommendations on radiological emergencies and malicious events could also take the following considerations into account:

1. Nuclear forensics and crime scene management is not only necessary in the context of law enforcement but should also generally be a part of radiation protection and is required immediately after the response.
2. Radiation protection demands an integrated approach considering the perspectives of experts of different fields – in the latter mentioned cases nuclear forensics, civil protection and law enforcement.
3. Consolidated mechanisms for multi-hazard emergencies are needed and need to be practiced regularly.
4. Emphasis could be put on cross-border arrangements (e.g. in the form of bilateral or multilateral agreements)
5. More advice on (medical) countermeasures is needed, the existing ones are too general in this respect.
6. Guidelines should be kept as simple as possible for emergency management.
7. Documents should be written and structured in a way that they can be directly translated into actions.

## Justification and optimisation of medical radiation applications.

There are three basic principles of radiological protection: justification, optimisation, and dose limitation. The latter, nevertheless, is not feasible in the field of medicine since there are no limit values for medical radiation exposure, i.e. for diagnostics or therapy. The principles of justification and optimisation should – in theory – be applied consecutively. In many civilian uses of ionising radiation this is not always applicable in practice, since “assessment of justification always involves some estimation on the dose, which is likely to already include some level of optimisation.” (NEA/CRPPH/R 2022). On the contrary, justification needs to maintain its central role in the medical field.

### Justification

The principle of justification of radiological procedures in medicine includes three levels (Level I-III). There are some considerations that should be taken into account. Level I justification (the use of radiation is doing more good than harm to society, i.e. the net benefit is positive) can be taken for granted in medicine (ICRP 2007, Sect.  7.1.). Level II justification (justification of a specified procedure with a specified objective) is crucial, when new technologies and or strategies are introduced (e.g. screening) or when individual justification is not possible or feasible (e.g. non-medical or medico-legal imaging). For level III justification (application of a specified procedure to an individual patient), the condition of the patient determines which modalities and procedures are appropriate. Also, the patient’s wishes and needs should be included by shared decision-making. Further, economic and social considerations must be implicated. Computed tomography (CT), for instance, is superior to radiography (Computed Radiography (CR)/Digital Radiography (DR)) but associated with higher exposure, thus making justification more critical for children. Magnetic Resonance Imaging (MRI) where appropriate to answer the diagnostic question, on the other hand, works without ionising radiation but is expensive, involves a long examination time and lacks of short term availability in emergency situations.

Justification should be and remain one of the central principles in medical applications of ionising radiation, followed by optimisation. There should be no exemptions to the principle of justification (e.g. for low-dose examinations). It is particularly important, that Level III justification (individual patient justification) is performed by persons with the proper qualification and requisite knowledge in radiation protection. This critical step cannot be delegated to the referring physician without proper qualification or skipped just because a referral has been issued.

### Optimisation

Optimisation is the other central principle of radiological protection in medicine and closely linked to the principle of justification. Different approaches are needed in diagnostics and therapy. In diagnostics, optimisation implies the attempt to obtain the best diagnostic information as possible with the lowest exposure. However, the exposure cannot be reduced continuously, as the image quality will deteriorate and the examination will not provide the relevant information of interest. This would result in an unnecessary exposure of the patient (and the personnel) to ionising radiation.

In therapy, the goal is to maximise the effect, i.e. to deliver sufficient dose to the tumour or the diseased tissue in order to eradicate the malignancy or heal it while keeping the doses to the surrounding healthy tissues as low as possible. This includes taking into consideration possible side effects like toxicity and tissue reactions, respectively, and late effects such as the possible induction of secondary cancers. Recently, increasing attention has been paid to the consequences of the effects of low doses received by the tissues even distant from the treatment site. In special cases, this consideration leads to an “as high as safely achievable” (AHASA) approach, when no other therapeutic options are available.

A sufficient level of quality is needed to ensure an accurate and reliable diagnosis or a successful therapy, respectively. Therefore, several considerations have to be taken into account. First, it is important to use up to date modalities, as far as available. Second, quality assurance (QA) as well as medical and technical aspects should be established and maintained. Third, doses should be optimised “as low as reasonably achievable” (ALARA). Different anatomical regions and patient characteristics should be noted, e.g. by the use of different protocols. Further, technological improvements should be applied, e.g. iterative reconstruction, or dose management systems. Also, patient specific optimisation should be utilised, e.g. CR/DR collimation, or field-of-view (FOV).

Optimisation can be further enhanced. Challenges in the daily clinical practice are given in two examples. First, diagnostic reference levels (DRL) have proven to be a powerful optimisation tool especially for radiological applications. However, their application in nuclear medicine has been less successful. Still open issues are the difficulty to set meaningful paediatric DRLs, due to the lack of homogenisation in weight and age categories and the (relative) scarcity of data, or the implementation of the DRL concept in the case of imaging for radiotherapy. A second challenge is the optimisation of the activity administered to paediatric patients in diagnostic nuclear medicine. Biokinetic data of children are rare and of variable quality, and in general it is necessary to scale the activities given to adults based on generic dosage cards, considering adult biokinetics and paediatric age-dependent mathematical phantoms. This not necessarily results in an optimised diagnostic protocol for paediatric patients.

### Dosimetry in nuclear medicine therapy

Dosimetry represents an important component of optimisation, especially in nuclear medicine. ICRP has published on dose coefficients for diagnostic radiopharmaceuticals (ICRP 1988, 1992, 1988, 2008a, 2015a). An update on the dose coefficients on radiopharmaceuticals used in diagnostic nuclear medicine was already published for public consultation. Radiation protection principles for radiopharmaceutical therapy were published by ICRP (ICRP 2019a). In the therapeutic uses individual dose assessments (i.e. not based on reference dose coefficients) are recommended. This has been included as a requirement in the recent Basic Safety Standard of the European Commission BSSD (Directive EC/2013/59/Euratom, http://data.europa.eu/eli/dir/2013/59/oj), which should be implemented in national regulations (e.g. the German Radiation Protection Act, StrlSchG, https://www.gesetze-im-internet.de/strlschg/ and Ordinance, StrlSchV, https://www.gesetze-im-internet.de/strlschv_2018/). One of the implementation issues in radiopharmaceutical therapy is that it combines two different legal fields (pharmaceutical and radiation protection) that might pose different and even conflicting requirements on the use of radiopharmaceuticals. The SIMPLERAD project (http://simplerad.eu) identified a lack of awareness of pharma regulators of the existence of the BSSD and a lack of intersection between European Medicine Agency’s (EMA) guidance documents (e.g. the future guideline: EMA/CHMP/451705/2024 (https://www.ema.europa.eu/en/documents/scientific-guideline/concept-paper-clinical-evaluation-therapeutic-radiopharmaceuticals-oncology_en.pdf) and radiation protection directives of the Directorate-General for Energy as main issues to be resolved in the implementation of a proper legal framework and regulatory procedures.

Individual dosimetry, which should be applied in clinical trials and clinical practice, needs to consider the radionuclide dependent nature of the emitted radiation, its associated dose rate and the spatially heterogeneous distribution of the radiopharmaceutical and its associated energy deposition. Many challenges are to be resolved to implement dosimetry in clinical practice, that include acquisition of suitable data, individual modelling and evaluation of results. Implementation of dosimetry in clinical workflows still is hampered by the lack of adequate resources. Methods of population modelling and small-scale biodistribution and dosimetry of the radiopharmaceuticals are recommended to be investigated in future by EURADOS (Bouwman et al. 2024; Li et al. 2022; Saldarriaga Vargas et al. 2024). As further individualisation of patient treatment will be needed, the European Association of Nuclear Medicine (EANM) recommended to increase teaching and standardisation and to incorporate radiobiology for improving the outcome and safety of patients undergoing radiopharmaceutical therapies (Lassmann et al. 2021). The Society of Nuclear Medicine and Molecular Imaging (SNMMI) updated its dosimetry primer to radiopharmaceutical therapy (Wahl 2021; MIRD 2022) and established Radiopharmaceutical Therapy Center of Excellence to provide the right treatment with the most advanced and evidence-based approaches tailored to the needs of each patient (SNMMI 2022). To fit the purpose, it is suggested that, in this drastic developing field of radiopharmaceutical therapy, ICRP might consider to initiate a long-term project not only about continuing the dose coefficients for diagnostic radiopharmaceuticals but also starting for the therapeutic radiopharmaceuticals in collaboration with other sister associations and societies.

### Summary

In summary, the combination of justification and optimisation is essential for medical radiation exposure and should be preserved. Optimisation is a continuous process which is also closely linked to technological advances. Continuous efforts towards optimisation can also result in the justification of procedures that were not justifiable before.

Changes in medical radiation protection have a big and immediate impact on a huge number of persons, mostly – but not limited to – patients. Therefore, medical radiation protection must remain in focus. As there are already many years of experience, principles and concepts reached already a high grade of maturity. Hence, it is less needed to introduce completely new concepts but rather to carefully develop further the existing system in order to adapt it to upcoming technologies and approaches in healthcare where appropriate.

The increase of the availability of medical imaging technologies and the development of new technologies in both diagnostics and therapy during the recent years, lead to an increased number of persons who have been exposed to radiation through medical imaging. Therefore, the application of justification and optimisation should derive even more importance and need to be an essential part of the qualification. Together with better treatments and a long-term survival rate the benefits and risks have to be reconsidered, and optimisation plays an increasingly important role.

Concerning dosimetry, there are many challenges in nuclear medicine. Dosimetry, especially individual dosimetry, must however be integrated into all processes and bring forth individual optimisation in order to keep up with future advancements.

## Impact assessment

The ICRP publication 103 of the International Commission on Radiological Protection (ICRP) from 2007 contains recommendations for maintaining the Commission’s fundamental principles of radiation protection as well as recommendations for a system change for radiation protection. The system of radiation protection described in ICRP publication 103 was the basis for the development of international standards (e.g. IAEA GSR Part 3 (European Commission et al. 2014)) and the European Directive 2013/59/Euratom, which was adopted into the German national radiation protection regulation (StrlSchG). Although the three fundamental principles of justification, dose limitation and optimisation were unchanged, the system for radiation protection changed fundamentally by the introduction of a situation-based approach that defines three categories of exposure situations: planned, emergency and existing exposure situations. Beside the changes of these fundamental principles ICRP publication 103 recommends further new measures and instruments, e. g. new measures for the protection against the exposure due to radon in dwellings and in indoor workplaces as existing exposure situations and the introduction of new instruments such as dose constraints in planned exposure situations and reference levels in existing exposure situations.

The current legal regulations for radiation protection in German radiation protection law offer an effective system to protect health while dealing with ionising radiation. However, the establishment of the new structure in the national regulations required lengthy and complex processes. Following publication of the Euratom Directive 2013/59/Euratom, the modernisation of the German radiation protection legislation was successfully completed in 2018, i.e. a good 10 years after publication of ICRP publication 103. Even 20 years after the publication of ICRP publication 103, the implementation of the legal requirements in enforcement as well as the development of corresponding sub-legal regulations is expected to be ongoing.

The implementation of international standards and the stabilisation of national regulations therefore takes time and there is a need for continued stability. It is therefore crucial that changes to the protection system, especially those that may lead to a change in the national legal framework, are justified with a clear, proportionate benefit that outweighs any disadvantages compared to the current system. This also applies when scientific knowledge has evolved and the underlying facts can be better and more realistically modelled. Consequently, ICRP should thoroughly consider the consequences of possible implementation for all proposed changes in terms of their integration into the regulatory framework before issuing revised recommendations.

## Overall conclusions

The revisions by the International Commission on Radiological Protection (ICRP) are crucial for advancing radiation protection standards. These updates integrate new scientific findings, societal perspectives, and improved methodologies into exposure and dosimetry calculations. The November 2024 workshop in Germany will help ensure that German expertise contributes to these global discussions, particularly in prioritizing key topics.

As radiation risks evolve, we must expand the focus beyond cancer and hereditary effects to include cardiovascular diseases and mental health concerns. This holistic approach will better align with modern societal needs. The growing evidence on circulatory diseases linked to low-dose radiation calls for updates in detriment calculations to reflect these emerging risks. Although hereditary effects remain a contentious issue, addressing them is key to improving the transparency and accuracy of current models.

Dosimetry remains foundational in radiation protection, and the ICRP's updated definitions and operational quantities will require careful recalculation and collaboration to avoid confusion. The shift in these definitions must ensure that safety measures remain intact and that personnel compliance is accurately monitored.

Incorporating social science perspectives into radiation protection is equally essential to improve public trust and risk communication. Mental health considerations, particularly during radiological emergencies, should be integrated into decision-making processes to enhance community resilience.

ICRP’s commitment to protecting non-human biota reflects a growing environmental concern, though effective and cost-efficient strategies are needed for practical implementation. Similarly, improving preparedness for radiological emergencies, including nuclear accidents, requires the development of reliable dosimetric tools, multi-hazard frameworks, and cross-border cooperation.

Finally, in medical radiation applications, justification and optimisation principles must evolve to ensure patient safety, particularly with emerging technologies. Overall, it is crucial that changes to the protection system, especially those that may lead to significant changes in the national legal frameworks, are justified with a clear, proportionate benefit that outweighs any disadvantages compared to the current system. Ensuring that changes to radiation protection systems are justified and proportionate is essential for maintaining an effective, science-backed framework.

The workshop organised by BMUKN and BfS in November 2024 is an important step in ensuring that German expertise is effectively represented in the international dialogue on these critical issues, particularly in relation to the prioritised topics selected for the workshop.

In summary, these developments underscore the need for an adaptive, interdisciplinary approach to radiation protection that ensures both scientific rigor and societal relevance.

## Recommendations at a glance


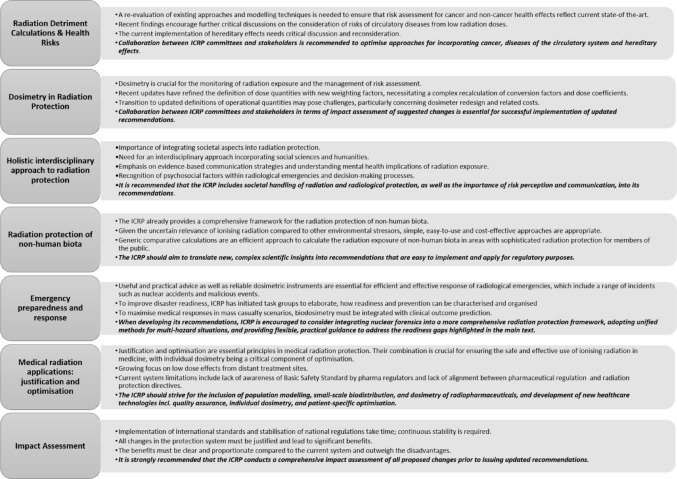


## Data Availability

No datasets were generated or analysed during the current study. Presentations of this workshop can be accessed via following link: https://www.bfs.de/EN/bfs/science-research/collaborations/workshop/workshop-icrp.html.

## Footnotes

1 The organisational decree issued by the Federal Chancellor on 6 May 2025 changed the structure and name of the ministry to BMUKN: Bundesministerium für Umwelt, Klimaschutz, Naturschutz und nukleare Sicherheit / Federal Ministry for the Environment, Climate Action, Nature Conservation and Nuclear Safety.

2 ProZES: https://www.bfs.de/EN/topics/ion/service/prozes/prozes.html

3 To provide a coarse estimate: The number of monitored workers in Germany is approx. 400,000. Worldwide ~ 20 Million workers are monitored. With an estimate of 2.5 dosimeters that are required per person to ensure a continuous monitoring and a cost of 10€ per device the required investments are 10 Mio € for Germany and 500 Mio€ worldwide. This doesn’t include the costs for R&D required to update the dosimeters.

4 RENEBRunning the European Network of Biological and Retrospective Physical Dosimetry https://www.reneb.net/
